# A Discrete Curvature Estimation Based Low-Distortion Adaptive Savitzky–Golay Filter for ECG Denoising

**DOI:** 10.3390/s19071617

**Published:** 2019-04-04

**Authors:** Hui Huang, Shiyan Hu, Ye Sun

**Affiliations:** 1 Department of Mechanical Engineering-Engineering Mechanics, Michigan Technological University, Houghton, MI 49931, USA; huih@mtu.edu; 2 School of Computer Science and Electronic Engineering, University of Essex, Colchester CO4 3SQ, UK; shiyan@mtu.edu

**Keywords:** ECG denoising, adaptive Savitzky–Golay filter, discrete curvature estimation, low distortion

## Abstract

Electrocardiogram (ECG) sensing is an important application for the diagnosis of cardiovascular diseases. Recently, driven by the emerging technology of wearable electronics, massive wearable ECG sensors are developed, which however brings additional sources of noise contamination on ECG signals from these wearable ECG sensors. In this paper, we propose a new low-distortion adaptive Savitzky-Golay (LDASG) filtering method for ECG denoising based on discrete curvature estimation, which demonstrates better performance than the state of the art of ECG denoising. The standard Savitzky-Golay (SG) filter has a remarkable performance of data smoothing. However, it lacks adaptability to signal variations and thus often induces signal distortion for high-variation signals such as ECG. In our method, the discrete curvature estimation is adapted to represent the signal variation for the purpose of mitigating signal distortion. By adaptively designing the proper SG filter according to the discrete curvature for each data sample, the proposed method still retains the intrinsic advantage of SG filters of excellent data smoothing and further tackles the challenge of denoising high signal variations with low signal distortion. In our experiment, we compared our method with the EMD-wavelet based method and the non-local means (NLM) denoising method in the performance of both noise elimination and signal distortion reduction. Particularly, for the signal distortion reduction, our method decreases in MSE by 33.33% when compared to EMD-wavelet and by 50% when compared to NLM, and decreases in PRD by 18.25% when compared to EMD-wavelet and by 25.24% when compared to NLM. Our method shows high potential and feasibility in wide applications of ECG denoising for both clinical use and consumer electronics.

## 1. Introduction

Electrocardiogram (ECG) sensing aims to record the electrical activities of the heart, which is performed by detecting minor electrical changes on the skin caused by heartbeat activities via electrodes. Since ECG signals intuitively represent the electrical activities of the human heart, ECG sensing becomes a useful diagnostic tool for detecting various cardiac problems [1,2]. Noise contamination is one of the challenges for accurate ECG sensing [3]. Due to the presence of various interferences during ECG sensing, ECG signals can be easily corrupted with a number of kinds of noise such as power-line interference, baseline wandering, respiration, muscle contractions, body movement, and noise from the acquisition devices. Recently, the emerging technologies of Wireless Body Area Network (WBAN) and pervasive healthcare enable low cost and ECG sensing via wearable sensor nodes and wireless transmission [4], which greatly extends the healthcare applications of ECG sensors in the diagnosis and preventive healthcare [5]. On the other hand, the process of ECG sensing can be contaminated by additional sources of noise such as the channel noise during wireless transmission, increased sensitivity to the disturbance in ECG sensors, and noise from the utilization of dry or non-contact electrodes [6]. In order to enhance the effectiveness of ECG sensors for accurate diagnosis, it is desired to develop new techniques for eliminating a number of kinds of noise from raw ECG signals.

ECG denoising has been investigated for decades in the literature and numerous methods have been developed. Conventionally, digital-filtering based denoising approaches are popular for ECG denoising. For example, several Kalman filtering based methods for ECG denoising are proposed with specific extensions for achieving good denoising performance [7,8,9]. Various adaptive filters [10,11] and Bayesian filtering based methods [12,13] are also developed for denoising ECG signals. In addition, approaches with other perspectives are proposed such as empirical mode decomposition (EMD) based [14,15] and discrete wavelet transform (DWT) based ECG denoising methods [16,17] which both discard the components of noise to estimate the clean ECG signal, ECG dynamical model (EDM) based methods which adopt nonlinear dynamical models for ECG signals denoising [18,19], and sample estimation based methods such as the non-local means (NLM) method [20] and the local polynomial regression method (LPR, also known as Savitzky–Golay (SG) Filter [21]), in which each sample is estimated based on specific criteria. For all these ECG denoising methods, they are quite effective in noise elimination for some scenarios. However, it is a challenge for these methods to handle the instantaneous high variations of ECG signals, which results in a trade-off between noise elimination and signal distortion.

For the denoising using an SG filter, a smooth polynomial representation is determined to estimate the local ECG signal, which is excellent for eliminating various kinds of noise, regardless of the properties of stationarity of the noise [22]. On the other hand, the SG filter has less adaptability to the signal variations as the SG filter requires a fixed polynomial order to compute the convolution weights for filtering. This issue particularly causes difficulties in ECG denoising due to the fact that ECG signals have high signal variations even in one R-R interval. That is, a high-order polynomial estimation of the local signal with small variation such as the flat parts of ECG signals will limit the performance for noise elimination as the high-order polynomials fit the noise samples as well, whereas a low-order polynomial estimation on dramatic variation such as the R peaks in ECG signals will result in significant distortion of the original signals.

In this study, instead of designing specific SG filters for applications, we propose an innovative and applicable low-distortion adaptive SG filtering (LDASG) method that is capable of self-adapting to input signals for ECG denoising. The proposed method utilizes adaptive polynomial order selection based on discrete curvature estimation of ECG signals. By estimating the curvature of each data sample in the discrete ECG signal, the signal variations can be reasonably represented and utilized for adaptively selecting the polynomial order of the SG filter. The proposed LDASG filter performs proper filtering processes according to the signal variations and thus achieves excellent performance for noise elimination as well as accurate reproduction of QRS complexes in the ECG signals. The main contributions of this study are summarized as follows:

- We develop a new LDASG filter for ECG denoising with adaptive polynomial order selection according to the variation of signals. Our method tackles the challenge of using a standard SG filter for denoising that has less adaptability to signal variations, resulting in difficulty of handling the instantaneous high variations of ECG signals and thus a trade-off of noise elimination and signal distortion.
- We propose to use the discrete curvature estimation to represent ECG signal variations and thus determine the order accordingly for adaptive SG filter design for each data sample. This newly proposed self-adaptive method maintains the intrinsic advantage of SG filter for noise elimination and tackles the difficulties due to the high variations of ECG signals even in one R-R interval.
- The experimental results of this study demonstrate that our method achieves excellent performance for ECG denoising and outperforms the other two state-of-the-art ECG denoising methods.

The remainder of this paper is organized as follows: Section 2 introduces the basis of the SG filtering theory. The details of the proposed method are given in Section 3. Section 4 presents the experimental results and the comparison with existing studies. A summary of the study is given in Section 5.

## 2. Fundamentals of the SG Filter

In this section, the theory of SG filter is first explained to better illustrate our proposed method. The SG filter approximates the underlying signal using local least square fitting and has remarkable performance for data smoothing problems, which derives directly from the time domain without having their properties defined in the frequency domain as general digital filters.

### 2.1. Basic Idea

**Figure 1:**
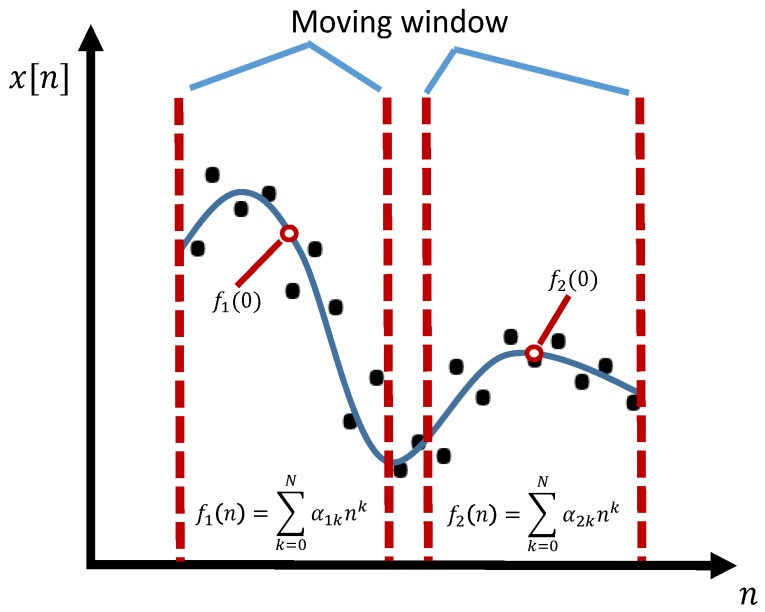
Illustration of the basic idea of SG filter.

The basic idea of SG filter is to approximate the underlying signal locally within a moving window using polynomial fitting with fixed order [21]. As shown in Figure 1, the coefficients of the polynomial can be determined in the least square sense and thus the smoothed data points are obtained by calculating the value of the polynomial at the center index of the moving window. For each data point, the filtered value is determined by repeating the above procedures.

Concretely, considering a symmetric window of 2M+1 samples centered at n=0,(−M≤n≤M where *M* denotes the half width of the window) within a sequence of signal x[n], the optimal polynomial fitting can be obtained as

(1) $$f\left(n\right)=\sum_{k=0}^{N}\alpha_{k}n^{k}$$

by minimizing the mean squared error (MSE) of the windowed samples with least square method as Equation (2),

(2) $$\epsilon =\sum_{n=-M}^{M}{\left(f\left(n\right)-x\left[n\right]\right)}^{2}=\sum_{n=-M}^{M}\left(\sum_{k=0}^{N}\alpha_{k}n^{k}-x\left[n\right]\right){}^{2},$$

where *N* is the order and $\alpha =\{\alpha_{k},k=0,1,\ldots ,N\}$ are the coefficients of the polynomial, respectively. The output data point is obtained by evaluating f(n) at the central point n=0 as Equation (3),

(3) $$y\left[0\right]=f\left(0\right)=\alpha_{0},$$

where *y* is the filtered signal. The value of the output data point is exactly the 0th polynomial coefficient in each moving window. The SG filter makes no use of the values of the fitting polynomial at any other point within the window. The next data point will be evaluated by the same procedures with performing a new least square polynomial fitting within a shifted window at the central location.

### 2.2. Methodology

It is laborious to perform polynomial fitting with least squares and evaluating the central location for each data point in the smoothing process of the original signal, although such a polynomial fitting and evaluating process has nothing to do with the shift invariant filtering. The original paper by Savitzky and Golay [21] proved that it is equivalent to performing a discrete convolution in a moving window as Equation (4),

(4) $$y\left[n\right]=\sum_{m=-M}^{M}h\left[m\right]x\left[n-m\right],$$

where h[m] is the fixed weighting coefficient. This is because the process of least square polynomial fitting is performed by linear matrix multiplication and inversion, which results in a linear relationship between the coefficients of the fitted polynomial and the value of the original data. More specifically, considering fitting a polynomial of degree *N* as Equation (1) within a window of length 2M+1, the design matrix A for this polynomial fitting problem is [22]

(5) $$A=\left\{A_{nk}\right\}=n^{k},-M\leq n\leq M,\;k=0,1,\ldots ,N.$$

Therefore, the coefficients of the fitted polynomial α using least square method can be evaluated by

(6) $$\alpha ={\left(A^{T}A\right)}^{-1}A^{T}x=Hx,$$

where $x={\left\{x\left[-M\right],\ldots x\left[0\right],\ldots x\left[M\right]\right\}}^{T}$ is the vector of the original data samples within the moving window and $H={\left(A^{T}A\right)}^{-1}A^{T}$ is a (N+1) by (2M+1) matrix. According to the previous discussion, the only concerned value of the fitted polynomial at n=0 is the output value, which is $y\left[0\right]=\alpha_{0}$. Therefore, only one row which corresponds to calculating $\alpha_{0}$ is necessary from matrix H. Since H is only determined by the order *N* and the half window width *M* and is independent from the input data samples in x[n], the corresponding row $H_{0}$ for computing $\alpha_{0}$ is fixed and considered to be the weighting coefficients of a shift invariant discrete convolution process as Equation (7),

(7) $$H_{0}=\left\{h_{0,-M},h_{0,-M+1},\ldots ,h_{0,0},\ldots ,h_{0,M}\right\}.$$

Consequently, the SG filtering is to apply the fixed weighted coefficients determined by the order of fitting polynomial and the width of the moving window to the discrete convolution of the original signal.

### 2.3. Application and Adjustment

SG filter has been tempted for various biomedical signal denoising [23,24,25]. In order to significantly improve the noise elimination performance with a low signal distortion for ECG signals, one remarkable study has been attempted to select the optimal length and order of the SG filter [26], in which the adaptive SG filter length and order selection methods are presented separately. The Steins unbiased risk estimator (SURE) was utilized to select the optimal length or order of the SG filter via near minimum mean squared error (MMSE). For ECG denoising, this method achieves good denoising performance. S. Rivolo successfully utilized the proposed algorithm in [26] for coronary wave analysis in [27]. For real-time processing or wearable devices, a more computation compact denoising method with superior performance is still highly desired.

## 3. Proposed LDASG Filter for ECG Denoising

As discussed in Section 1, the standard SG filter has less adaptability on the signal variations. For ECG signal denoising, the wide range of signal variations imposes a trade-off between the noise elimination performance and the signal distortion on the standard SG filter. In order to significantly improve the noise elimination performance with a low signal distortion for ECG signals, it is desirable to adapt the SG filter to the signal variations by varying the fitting polynomial order. Therefore, a new approach of adaptive SG filter is proposed and the details of the proposed method are explained in the following sections.

### 3.1. Overview

**Figure 2:**
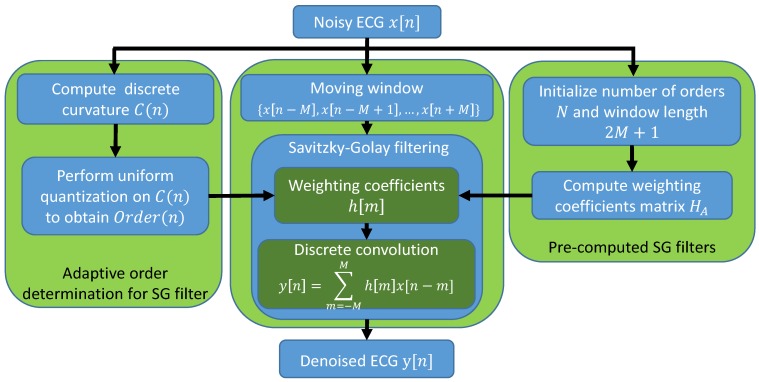
System architecture of the proposed LDASG filter for ECG signal denoising.

In our approach, discrete curvature is introduced for the one-dimensional time-varying ECG signals to quantitatively represent the signal variations. The estimation of discrete curvature on each data sample is utilized to select the polynomial order of the SG filter adaptively. As shown in Figure 2, the discrete curvature of each data point of the noisy ECG signal is calculated and further utilized for determining the order of the SG filter by using uniform quantization. The weighting coefficients of the SG filters with different orders are pre-computed according to the initialized window length 2M+1 and the total number of orders *N*. Therefore, for the filtering process, instead of using a fixed SG filter, each data point is filtered by the proper SG filter based on its discrete curvature, which eliminates the noise more precisely and lowers the signal distortion during the filtering process.

### 3.2. Discrete Curvature Estimation for One-Dimensional Time Series

Discrete curvature is an important geometrical parameter in various applications of computer vision and digital imaging. In our work, the time series of ECG signals can be considered as planar curves, which are uniquely determined by their curvature profiles [28]. Therefore, the signal variations of ECG signals can be represented quantitatively by the discrete curvature. Although the curvature of a smooth planar curve is clearly defined in the theory of differential geometry, the estimation of the curvature of discrete curves with noise is a nontrivial task. This is because the continuous representation of the discrete curves is unavailable and the singular data points of noise make the curvature calculation highly unstable and therefore result in exponential errors [29]. Despite the challenges above, numerous discrete curvature estimation approaches are proposed [30,31,32]. Unlike the standard discrete curvature estimation which considers the estimation of curvature in two dimensions, the discrete curvature estimation for time series only concerns one dimension of data samples as one of the dimensions is evenly distributed and excluded from noise contamination. In this study, a one-dimensional discrete curvature estimation method simplified from standard discrete curvature estimation methods [33] is used for the curvature estimation of time-varying ECG signals. The one-dimensional discrete curvature estimation method is based on the variation of tangent, which is approximated by the longest digital straight segment (DSS) of data samples. Considering both sides of a data point, two DSS are estimated as well as their tangents. The variation between the two tangents are used for computing the curvature of the considered data point.

#### 3.2.1. Definitions

For each data point $p_{i}=\left(t_{i},x_{i}\right)$ in the time series, the forward and backward searching vectors are defined as Equation (8),

(8) $$\begin{array}{c} \mathrm{forward}\;\;\mathrm{vector}\;f_{i,k}=p_{i}-p_{i+k},\\ \mathrm{backward}\;\;\mathrm{vector}\;b_{i,k}=p_{i}-p_{i-k}, \end{array}$$

where *i* is the index of data sample, and *k* is the searching length for the DSS. The estimated slope angle of the tangent line at points $p_{i}$ is defined as

(9) $$\theta_{i,k}=\tan^{-1}\left(\right|\frac{x_{i}-x_{i-k}}{t_{i}-t_{i-k}}\left|\right)$$

and the centered slope angle variation of data point $p_{i}$ is defined as

(10) $$\delta_{i,k}=\theta_{i+1,k}-\theta_{i-1,k}.$$

#### 3.2.2. Discrete Curvature Estimation

The discrete curvature of the data point is estimated by the slope angle variation between the forward and backward longest DSS. The first step is to determine the longest DSS according to a well-designed criterion [34]. Consider a small angle variation Δ, which can be used to represent the reasonable deviations of a sequence of data points due to noise. If any of the centered slope angle variation of each data point in the sequence is equal or less than Δ, the sequence of data points can be considered as the DSS. The maximum searching vector length can be evaluated as Equation (11):

(11) $$\begin{array}{c} k_{b}=max\left\{k:\forall s\left(1\leq s\leq k\to -\Delta \leq \delta_{i-s,2}\leq \Delta \right)\right\},\\ k_{f}=max\left\{k:\forall s\left(1\leq s\leq k\to -\Delta \leq \delta_{i+s,2}\leq \Delta \right)\right\}, \end{array}$$

where $k_{b}$ and $k_{f}$ are the maximum length of the backward and forward searching vector for DSS. Here, the centered slope angle variation is based on the distance of 2 denoted by $\delta_{i,2}$, which means the slope angle of the tangent line of the data point is computed with the second neighbor. Accordingly, the length of the longest DSS can be calculated by

(12) $$\begin{array}{c} L_{b}=\sqrt{{\left(x_{i}-x_{i-k_{b}}\right)}^{2}+{\left(t_{i}-t_{i-k_{b}}\right)}^{2}},\\ L_{f}=\sqrt{{\left(x_{i}-x_{i+k_{f}}\right)}^{2}+{\left(t_{i}-t_{i+k_{f}}\right)}^{2}}, \end{array}$$

where $L_{b}$ and $L_{f}$ are the lengths of backward and forward DSS, respectively. In addition, the angles of the two DSS are calculated by

(13) $$\begin{array}{c} \theta_{b}=tan^{-1}\left(\right|\frac{x_{i}-x_{i-k_{b}}}{t_{i}-t_{i-k_{b}}}\left|\right),\\ \theta_{f}=tan^{-1}\left(\right|\frac{x_{i}-x_{i+k_{f}}}{t_{i}-t_{i+k_{f}}}\left|\right), \end{array}$$

where $\theta_{b}$ and $\theta_{f}$ are the slope angles of the backward and forward DSS. Finally, the curvature estimation at data point $p_{i}$ is as Equation (14) [32]

(14) $$C_{i}=\frac{\left(L_{b}+L_{f}\right)\left(\theta_{b}+\theta_{f}\right)}{4L_{b}L_{f}}.$$

**Figure 3:**
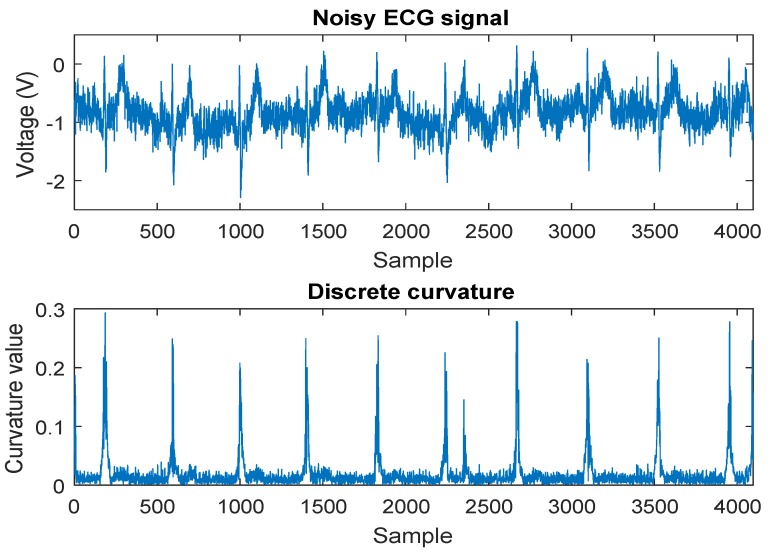
Discrete curvature of a noisy ECG signal.

The procedures of the discrete curvature estimation are presented in Algorithm 1. Figure 3 shows the discrete curvature estimation for a noisy ECG signal. According to Figure 3, the values of the discrete curvature are consistent with the data points at peaks and the flat parts of the noisy ECG signal, which indicates that the signal variations can be reasonably represented by the discrete curvature. In addition, the experimental results in Section 4 demonstrate the reliability of the discrete curvature estimation.
**Algorithm 1** Discrete curvature estimation for time seriesInitialize a maximum length of the searching vector $k_{m}$ and a small angle variation Δ, within the range of which a sequence of data points can be considered as a DSS;Find the maximum length of the backward searching vector for data point $p_{i}$. For the data points from $p_{i-k}$ to $p_{i-1}$, compute and check if all their centered slope angle variations satisfy $-\Delta \leq \delta_{j,2}\leq \Delta \left(i-k\leq j\leq i-1\right)$. If the condition does not meet, $k_{b}=k$. If $k\geq k_{m}$, $k_{b}=k_{m}$;Find the maximum length of the forward searching vector for data point $p_{i}$. For the data points from $p_{i+1}$ to $p_{i+k}$, compute and check if all their centered slope angle variations satisfy $-\Delta \leq \delta_{j,2}\leq \Delta \left(i+1\leq j\leq i+k\right)$. If the condition does not meet, $k_{f}=k$. If $k\geq k_{m}$, $k_{b}=k_{m}$;Compute the length of the backward and forward DSS, $L_{b}$, $L_{f}$, and the slope angles of the two DSS, $\theta_{b}$, $\theta_{f}$.Compute the curvature as $C_{i}=\frac{\left(L_{b}+L_{f}\right)\left(\theta_{b}+\theta_{f}\right)}{4L_{b}L_{f}}$.

### 3.3. Proposed LDASG Filter

The proposed LDASG filter features in adaptively selecting the order of the fitting polynomial according to the value of the discrete curvature of the data point. In our method, the uniform quantization process of the discrete curvatures of each data point is leveraged for mapping the discrete curvature to a sequence of orders of the fitting polynomial from 1 to *N* as follows:

(15) $$Order\left(n\right)=floor\left(\frac{NC(n)}{C_{max}-C_{min}}+\frac{1}{2}\right),$$

where $C_{max}$ and $C_{min}$ are the maximum and minimum value of the estimated curvature, floor(z) is the floor function which gives the greatest integer less than or equal to *z*. Since the order selection for each data sample is based on the quantization process of the discrete curvature, an important consideration is the total number of orders *N*. Although there are various mapping processes for mapping the discrete curvature to the sequence orders from 1 to *N*, we experimentally observed that the uniform quantization with 9≤N≤20 are sufficient for ECG denoising by our method.

Therefore, for each data point, a proper order is selected from the pre-defined sequence of orders for constructing the SG filter. Figure 2 shows the system architecture of the proposed LDASG filter with the adaptive order determination and pre-computed initialization. The length of the moving window of the SG filter 2M+1 is fixed for all the data samples, which is initialized empirically depending on the sampling rate of the ECG signals as well as the noise power. We also attempt varying window length in the experiment and found that results show negligible variations of the performance. Thus, we do not include the details in the paper. The window length can be initialized or selected as in the standard SG filter. With the sequence of *N* orders and the length of window 2M+1, *N* number of SG filters are constructed as well as the corresponding weighting coefficients of the equivalent discrete convolution, which is the matrix for the adaptive SG filtering denoted by

(16) $$H_{A}={\left\{H_{0}^{1},H_{0}^{2},\ldots ,H_{0}^{N}\right\}}^{T},$$

where $H_{0}^{i},1\leq i\leq N$ is the corresponding weighting coefficients. Consequently, the adaptive SG filtering for the ECG signals is to apply the discrete convolution for each data point using the corresponding weighting coefficients in $H_{A}$. The details of the method are included in Algorithm 2.

**Algorithm 2** LDASG filtering for ECG denoising
Initialize the number of orders *N* and the length of moving window 2M+1;Compute the discrete curvature C(n) of the input ECG signal x[n] using Algorithm 1, except the data points in the first and last window;Find the maximum and minimum value in C(n) and perform uniform quantization to map C(n) to Order(n) via Equation (15);Compute the weighting coefficients matrix $H_{A}$ of SG filters according to the length of moving window 2M+1 and orders *N*;For the data points within the first and last window, they are replaced by the value of fitting polynomial of the SG filter on the central data point. Perform discrete convolution on the rest input ECG signal x[n] via Equation (4), the weighting coefficients h[m] for each data point are determined by the Order(n) row of $H_{A}$;The output of the discrete convolution y[n] is the result of the adaptive SG filtering.


### 3.4. The Algorithm

From the discussions of the above two sections, the first step of the algorithm of the LDASG filtering method for ECG denoising is the initialization of parameters including the maximum length of searching vector $k_{m}$ and the small angle variation Δ for the discrete curvature estimation, and the number of orders *N* and the length of moving window 2M+1 for the LDASG filtering. The next step is to compute the discrete curvature, C(n), of each data point for determining the order, Order(n), of the SG filtering by uniform quantization, and the weighting coefficients matrix, $H_{A}$, of all the SG filters according to the initialized number of orders. The final step involves the filtering process which is the discrete convolution with the adaptively determined weighing coefficients h[m] from $H_{A}$. The output of the discrete convolution is the denoised ECG.

## 4. Experiments and Results

**Figure 4:**
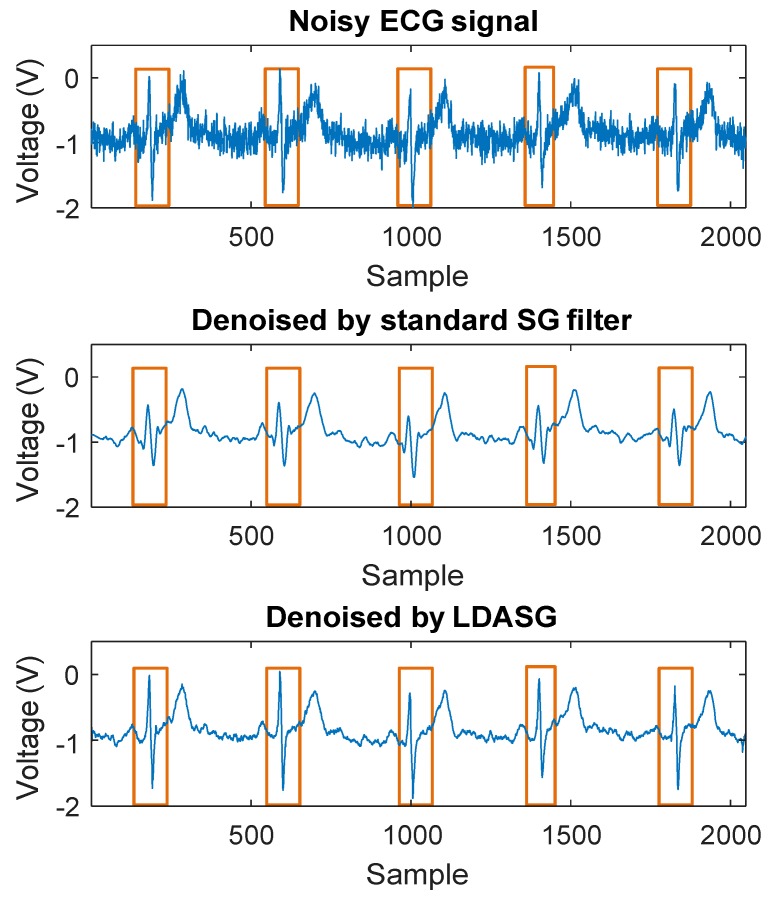
Denoising results of standard SG filtering and the proposed method. With comparable noise elimination performance, the standard SG filter has greater distortions at high variation parts, especially at R peaks, than the proposed method.

In this section, the denoising performance of the proposed method is evaluated. Figure 4 shows the results of the standard SG filtering with properly selected window length and order and the proposed LDASG filtering method on a real wearable noisy ECG signal. From Figure 4, the standard SG filter achieves comparable noise elimination with the proposed method while the distortions at the high variations, especially the R peaks, are significantly greater than the proposed method. Next, real ECG data and simulated noise with controllable SNR levels are used for the evaluation and comparison with the state-of-the-art methods of our method.

### 4.1. Real ECG Database with Artificial Contamination

**Figure 5:**
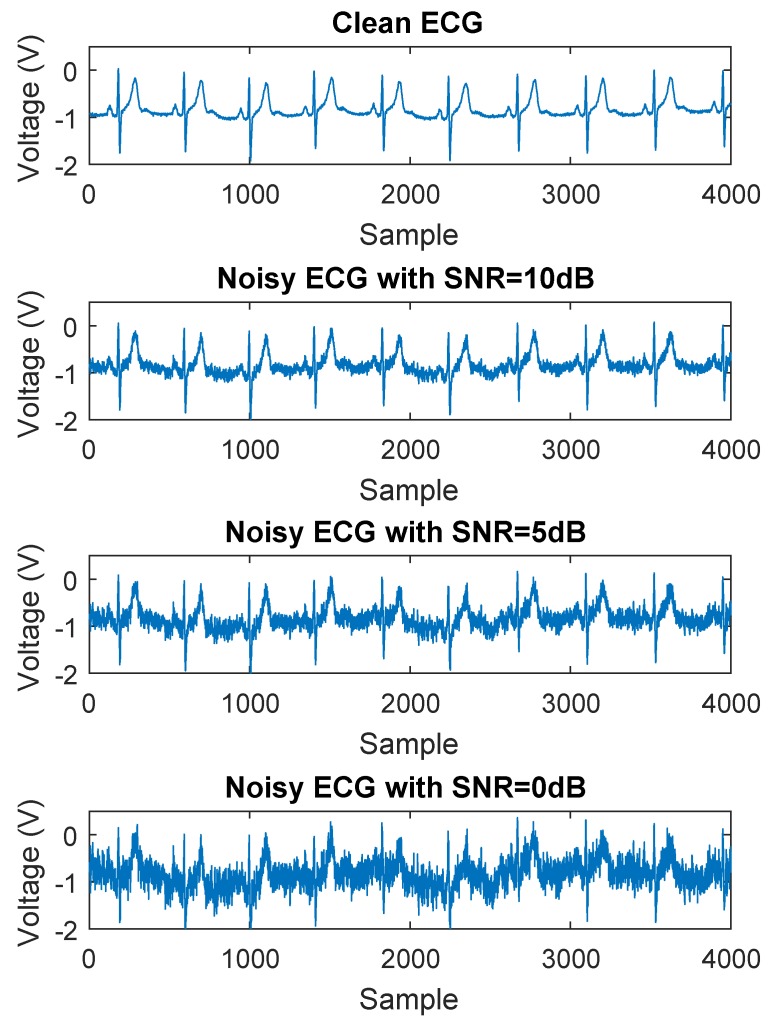
Artificially contaminated ECG signals with different SNR levels.

In order to thoroughly validate the performance of the proposed method and compare with existing studies quantitatively, the widely used ECG database, MIT-BIH arrhythmia database [35], is used with artificial contamination for creating various noisy ECG signals. The noisy ECG signals are created by adding various noise, such as White Gaussian Noise (WGN), nonstationary real Muscle Artifacts (MA) noise and colored noise, to the clean ECG signals. In this way, the noise can be scaled and combined to generate a range of SNR levels for performance evaluation, which is widely used by other ECG denoising methods for quantitative evaluation [9,10,11,20]. As not all the ECG signals in the database are clean enough, the following ECG records in the database are selected as the source of clean ECG signals: 101, 103, 104, 105, 106, 115, 117. To simulate common noise in ECG signals, two kinds of synthetic noise, WGN and pink noise, and a real MA from MIT-BIH Noise Stress Test Database [35] are considered in our experiment due to the comparable power spectrum to realistic ECG noise. The pink noise and WGN are created according to the power spectral density as

(17) $$S\left(f\right)\propto \frac{1}{f^{\beta }},$$

where *f* is the frequency, S(f) is the power spectral density function, β indicates the type of noise with β=0 for WGN and β=1 for pink noise. The three types of noise are combined and scaled to different SNR levels for performance evaluation in the experiments. Figure 5 shows the artificially contaminated ECG signals with different SNR levels. In this figure, the original ECG record from the database is considered to be clean, though it contains noise theoretically. Three levels of SNR are selected to generate noise and illustrate the denoising performance, 10 dB, 5 dB, and 0 dB. With the SNR of 10 dB and 5 dB, the QRS complexes are still visible; whereas with the SNR of 0 dB, the clean ECG is almost immersed in the noisy signal.

### 4.2. Performance Metrics

ECG signal denoising methods are typically evaluated based on the capability of eliminating noise with low signal distortion [9,10,11,20]. According to the literature, three metrics for the evaluation are widely used. The first is SNR improvement ($SNR_{imp}$) which represents the signal quality improvement over noise. The second is MSE which signifies how close it is between the clean and denoised signals. The third is PRD which indicates the percentage distortion of the denoised signal. The three metrics can be expressed mathematically as follows:

(18) $$SNR_{imp}=10log_{10}\frac{\sum_{k=1}^{K}x^{2}\left[k\right]}{\sum_{k=1}^{K}{\left(y\left[k\right]-x\left[k\right]\right)}^{2}},$$

(19) $$MSE=\frac{1}{K}\sum_{k=1}^{K}{\left(y\left[k\right]-x\left[k\right]\right)}^{2},$$

(20) $$PRD=100\sqrt{\frac{\sum_{k=1}^{K}{\left(y\left[k\right]-x\left[k\right]\right)}^{2}}{\sum_{k=1}^{K}x^{2}\left[k\right]}},$$

where x[k] is the original clean ECG signal, y[k] denotes the denoised ECG signal and *K* is the total number of samples of the ECG signal.

### 4.3. Experimental Results

**Figure 6:**
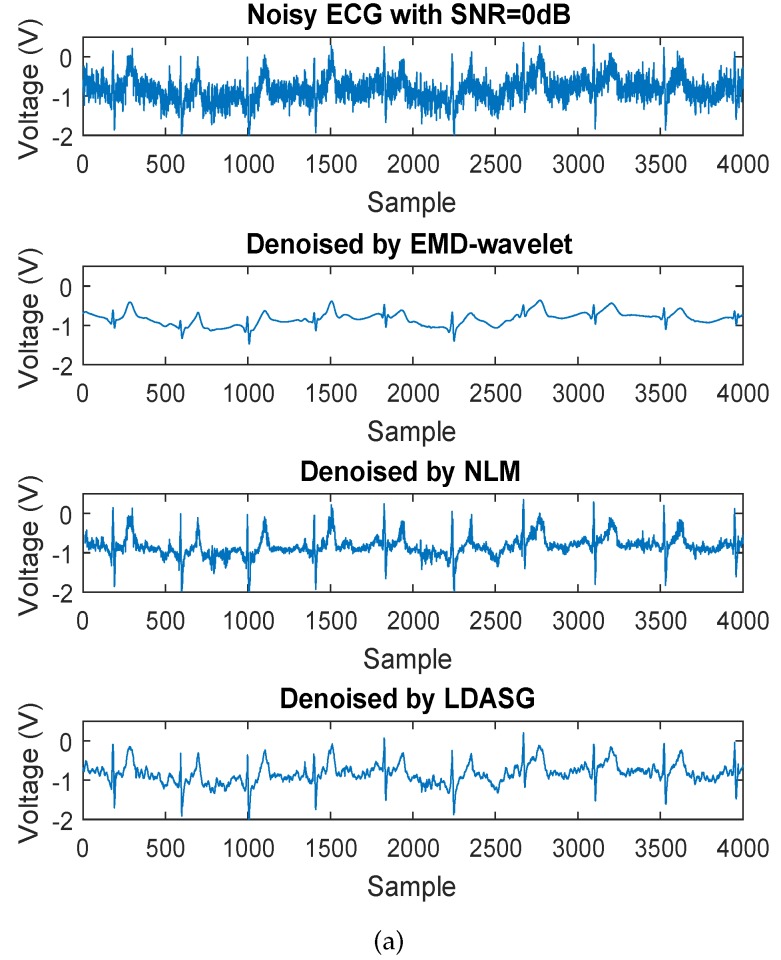

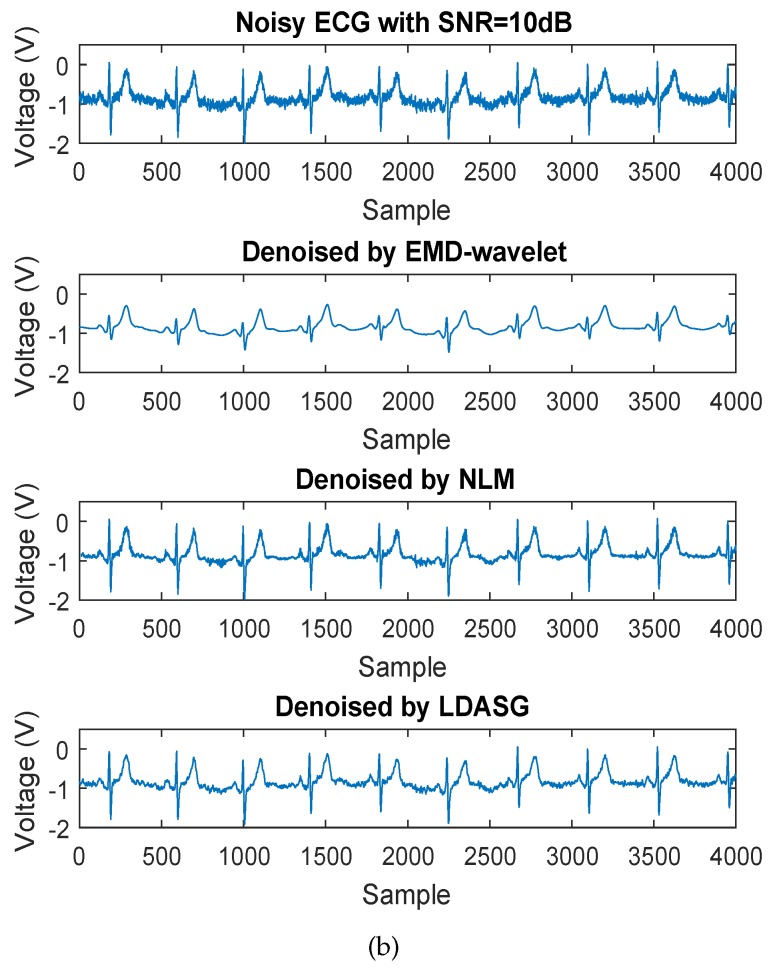
Denoising results comparison of the three methods. (**a**) Results on the noisy ECG signal with the SNR of 0 dB; (**b**) Results on the noisy ECG signal with the SNR of 10 dB.

The proposed method is evaluated on various noisy ECG signals with a range of SNR levels from 0 dB to 10 dB. For comparison with existing studies, two benchmark ECG denoising methods, the EMD-wavelet based method [15] and the non-local means (NLM) denoising method [20], are implemented and performed on the noisy ECG signals in our experiments, as they are reported to outperform the traditional methods such as wavelet-transform based ECG denoising methods. Figure 6 shows the denoising results of the above methods on a noisy ECG signal with the SNR of 0 dB and 10 dB. The parameters of EMD-wavelet and NLM methods are determined according to [15,20], respectively. From Figure 6, all of the three methods are able to handle non-Gaussian and nonstationary noise. It is noticed that the result of EMD-wavelet method seems to have a good performance of smoothing the noisy ECG signal while the signal distortion is greater than the other two methods as some of the peak values are greatly reduced. The results of the other two methods have less signal distortion after denoising and the proposed method has better smoothing performance visibly.

**Figure 7:**
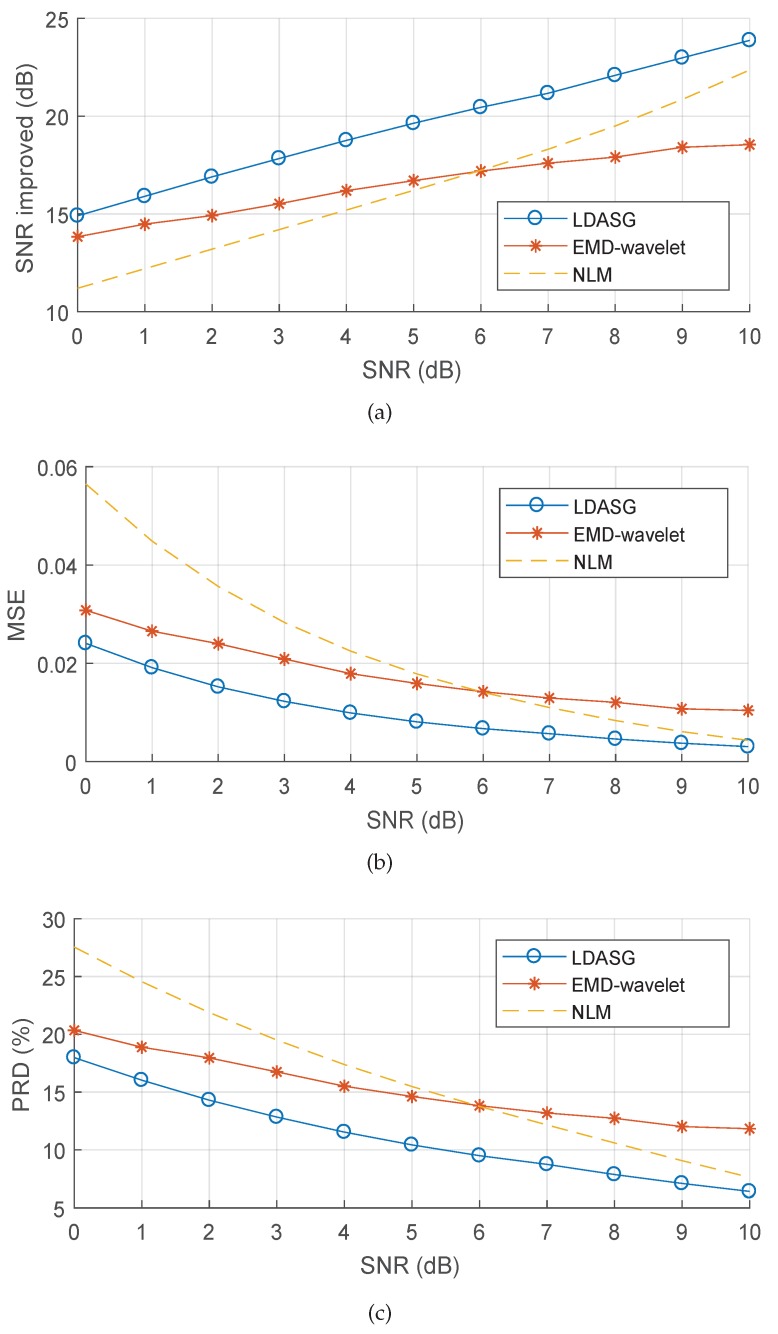
Average ECG denoising performance of the three methods on different noisy ECG signals with the SNR level from 0 dB to 10 dB. (**a**) SNR improvement; (**b**) MSE; (**c**) PRD.

For the quantitative evaluation and comparison, the average performances of the methods on different noisy ECG signals at a range of SNR levels are shown in Figure 7. According to Figure 7a, the NLM method has higher SNR improvements than EMD-wavelet method when the SNR of the noisy ECG signal is higher than 6 dB. Our proposed method achieves greater SNR improvements for the noisy ECG denoising in both high and low noise level condition with the SNR of 0–10 dB, which outperforms the other two existing methods in the view of SNR improvement and thus demonstrates better noise eliminating capability. For the signal distortion, as Figure 7b,c shows, the MSE and PRD of the EMD-wavelet method are higher than the NLM method when the SNR of the noisy ECG signal is higher than 6 dB, while they are much lower when the SNR decreases to 0 dB. The MSE and PRD of our method are lower than both of the two methods in the SNR range of 0–10 dB, which indicates that it achieves less signal distortion than the other two methods. Particularly at low SNR levels, it is noticed that the proposed method has much more improvement for lowering signal distortion. With the SNR of 0 dB, the SNR improvement of the proposed method is slightly greater than the other two methods while the MSE and PRD of the proposed method are substantially less than that of the other two methods. For a detailed demonstration, Table 1 shows the performance comparison among the three methods on individual ECG signals with the SNR of 0 dB. From the table, the MSE and PRD of the proposed method on most of the ECG signals are much less than the other two methods. For the averaged performance, our method decreases in MSE by 33.33% when compared to EMD-wavelet and by 50% when compared to NLM, and decreases in PRD by 18.25% when compared to EMD-wavelet and by 25.24% when compared to NLM, which demonstrates the superiority of the proposed method on low signal distortion at low SNR levels. Considering the computation, most of the computation costs of the proposed method are mainly in the discrete curvature estimation. Since the discrete curvature estimation involves the calculation of several equations from (12) to (14) within a limited search range, the computation costs will not be as high as solving an optimization problem like that in [26]. In order to investigate the computation complexity of the proposed method, Table 2 presents the computation times of denoising a 20 s ECG signal in MATLAB with an Intel Core i7 3.2GHz processor and 16 GB RAM. From the table, the computation time of our method is less than that of the EMD-wavelet method and greater than that of the NLM method. The results show that our method has comparable computation complexity with the state-of-the-art methods.

The reason for the superior ECG denoising performance of the proposed method lies on the adaptive order selection of SG filter based on discrete curvature. SG filters have an excellent performance for smoothing data, which makes it advantageous for eliminating relatively high frequency noise. As ECG signals have dramatic signal variations, it is difficult to eliminate the noise without signal distortion using a single SG filter. In order to reduce the signal distortion, the discrete curvature is introduced to represent the signal variations for designing proper SG filters adaptively. Therefore, coupled with the two advantages, the proposed method is able to achieve better performance of both noise elimination and low signal distortion for ECG denoising.

### 4.4. Experiments and Results on Real Wearable ECG Signals

**Figure 8:**
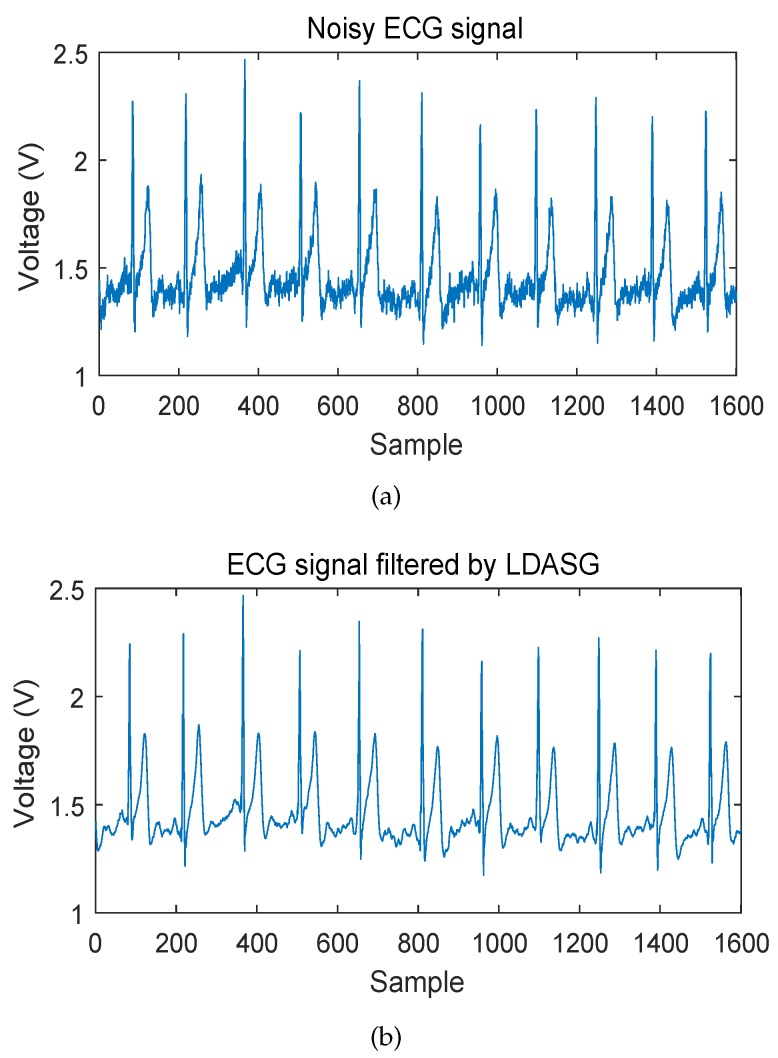
Result of denoising real wearable ECG signal by LDASG. (**a**) noisy ECG signal; (**b**) denoised ECG signal.

Based on the validation and simulation on the existing database to compare with the state of the art, we use the proposed method further in practical studies. The ECG data were acquired from a wearable ECG sensor with a non-contact electrode developed by our previous study. Due to various noise contamination, the ECG signal is noisy as shown in Figure 8a. We then applied our proposed LDASG method on these ECG recordings and validated the performance visually. Figure 8b shows the result of the denoising by LDASG. The noisy ECG signal can be smoothed well with negligible distortion.

## 5. Conclusions

In this paper, a new LDASG filter based on discrete curvature estimation is developed for ECG denoising. In order to retain the excellent smoothing performance of SG filters and tackle the challenge of mitigating the signal distortion for denoising high variation signals, the discrete curvature estimation is introduced for representing the signal variations and adaptively designing proper SG filters for each data sample. The proposed LDASG filter combines the advantages of excellent smoothing performance and low signal distortion and achieves promising performance of ECG denoising. The performance is evaluated by comparing with the state of the art, i.e., two benchmark ECG denoising methods using real ECG signals. Our method achieves much lower MSE and PRD than the other two methods. The experimental results demonstrate that our method shows better noise eliminating capability and lower signal distortion. In addition, in the situation of high noise power (low SNR), our method achieves significantly better performance of lowering the signal distortion for ECG denoising. The proposed method provides a promising solution for various applications of ECG denoising in both clinical use and wearable technologies.

## Figures and Tables

**Table 1:** Results of ECG denoising performance with the SNR level of 0 dB.

	SNR Improvement			MSE			PRD (%)		
ECG Records	EMD-w.	NLM	LDASG	EMD-w.	NLM	LDASG	EMD-w.	NLM	LDASG
#101	9.5	9.05	10.47	0.015	0.017	0.012	33.48	35.29	29.96
#103	7.16	7.83	10.35	0.029	0.026	0.014	43.87	40.61	30.37
#104	8.85	7.79	10.39	0.016	0.021	0.011	36.09	40.88	30.21
#105	9.71	8.22	10.78	0.015	0.022	0.011	32.71	38.83	28.91
#106	6.79	6.16	9.45	0.041	0.048	0.022	45.73	49.23	33.69
#115	8.06	7.29	9.17	0.051	0.061	0.039	39.55	43.16	34.78
#117	13.85	11.24	14.91	0.031	0.056	0.024	20.28	27.41	17.98
Average	9.13	8.23	10.79	0.03	0.04	0.02	35.96	39.34	29.41

**Table 2:** Comparison of the computation time of different methods (seconds).

ECG Reconds	EMD-Wavelet	NLM	LDASG
#101	0.792	0.490	0.550
#103	0.730	0.490	0.531
#104	0.767	0.509	0.583
#105	0.815	0.469	0.582
#106	0.754	0.507	0.459
#115	0.789	0.507	0.569
#117	0.779	0.474	0.622
Average	0.775	0.492	0.556
